# Analyzing Mega-mobility Systems in Smart Cities: A Macro–Micro Integration with Feedback Paradigm Empowered by Artificial Intelligence

**DOI:** 10.34133/research.0982

**Published:** 2025-12-04

**Authors:** Zelin Wang, Qixiu Cheng, Ziyuan Gu, Chengqi Liu, Dongyue Cun, Xinyu Shi, Xihan Wu, Zhen Zhou, Xiangyu He, Ljubo Vlacic, Zhiyuan Liu

**Affiliations:** ^1^ Jiangsu Key Laboratory of Urban ITS, Jiangsu Province Collaborative Innovation Center of Modern Urban Traffic Technologies, School of Transportation, Southeast University, Nanjing, China.; ^2^ University of Bristol Business School, University of Bristol, Bristol, UK.; ^3^ College of Civil and Transportation Engineering, Hohai University, Nanjing, China.; ^4^ Institute of Intelligent & Integrated Systems and the School of Engineering and Built Environment, Griffith University, Brisbane, Australia.

## Abstract

As pivotal drivers of smart cities, mega-mobility systems integrate large-scale transportation networks, communication nodes, and energy circuits into a coupled multinetwork system. Urban megasystems epitomize the grand challenge of “organized complexity”, exhibiting characteristic features such as adaptive openness, nonlinear dynamics, hierarchical organization, and emergent properties. Analytical investigations, constrained by the rigid separation of macro- and microlevel paradigms, struggle to capture the nonlinear interdependencies across levels that define mega-mobility systems. In this review, we systematically advance macro–micro integration with feedback (MMIF) as a transformative paradigm for analyzing urban mega-mobility systems, synthesizing the state-of-the-art developments in typical constituent subsystems under this unified perspective. The MMIF paradigm bridges the gap between theoretical abstraction and empirical practice, contributing to scientifically sound urban development by harmonizing emergent patterns with granular behavioral dynamics. Building upon this paradigm, we investigate the key methods and technologies empowered by artificial intelligence that enable MMIF and critically analyze the enduring challenges and prospective research directions. As urban mobility systems increasingly serve as test beds for complexity science, the MMIF paradigm using artificial intelligence promises to reshape interdisciplinary collaboration, offering a blueprint for building intelligent, adaptive, and human-centric cities.

## Introduction

**Fig. 1.**
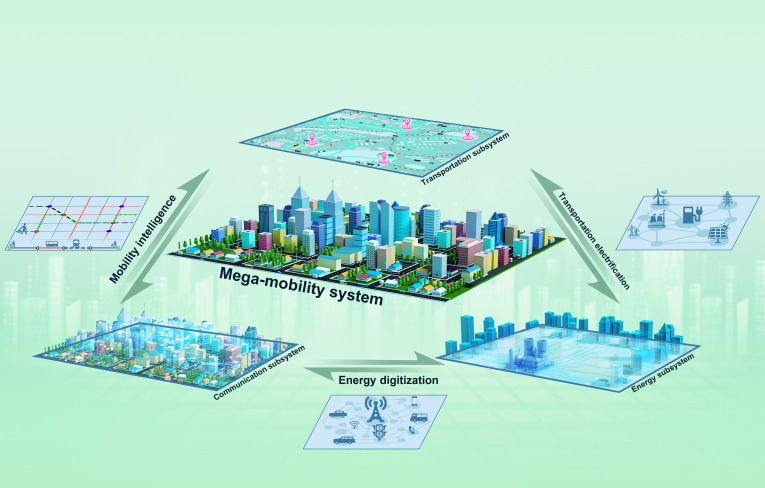
Mega-mobility system in cities with its typical constituent subsystems.

Warren Weaver’s systematic categorization of “organized complexity” and Philip W. Anderson’s seminal proclamation that “more is different” catalyzed the emergence of complex systems, a transdisciplinary paradigm investigating how microscopic interactions generate macroscopic phenomena [1]. This paradigm shift has found a highly practical application in the study of urban mobility systems, a key area of social development (see Fig. 1). Mega-mobility systems, characterized by human activities, are archetypal nonlinear megasystems integrating large-scale transportation networks, communication nodes, and energy circuits in a deeply coupled multinetwork structure [2]. As Stephen Hawking observed, the 21st century heralds the era of complexity science [3], and mega-mobility systems stand as both a theoretical laboratory for exploring irreducible complexities and a frontier of smart city evolution [4].

Current analytical approaches to urban mobility systems remain bifurcated into macroscopic and microscopic methodologies, each with distinct epistemological foundations and practical limitations [5]. Macroscopic analyses adopt a top-down perspective to capture city-scale phenomena. Systems science, from this viewpoint, involves global observation across multinetwork layers. Its core philosophy is rooted in emergence theory: when the collective behavior of system components exceeds critical thresholds, discrete interactions coalesce into novel qualitative features that go beyond simple linear aggregation [6]. This essential property of “the whole being greater than the sum of parts” drives researchers into statistical mechanics and network topology analysis. In contrast, the microscopic perspective originates from adopting bottom-up reductionist strategies to decode system fundamentals. Its methodological framework focuses on constructing elemental dynamical equations, rigorously describing individual state transitions through Markov chains, strategy-updating rules in game theory, or potential field equations in multibody systems, thereby elucidating the microfoundations of emergent collective behaviors [7]. This approach prioritizes system heterogeneity, which includes spatial correlations in particle clusters, the fractal characteristics of behavioral preferences, and the propagation modes of nonequilibrium fluctuations. These elements form the basic units for understanding systemic evolution [8]. The dual perspectives in studying mobility systems resemble the wave–particle duality in quantum mechanics, forming inseparable complementary projections within the cognitive continuum, each perspective capturing essential aspects of reality that become mutually exclusive when observed through singular frameworks, yet inherently unified in the object of study.

To bridge this scale divide, multiscale modeling [9] (which relies on one-way information flow—either bottom-up parameter passing or top-down field imposition) and hybrid-fidelity modeling [10] (which integrates high- and low-accuracy models to balance cost and precision) have each emerged as powerful frameworks. However, they often operate in isolation. Multiscale methods struggle to manage model uncertainty, and hybrid-fidelity approaches may ignore nonlinear feedback across scales. Macro–micro integration with feedback (MMIF) combines these strengths by embedding hybrid-fidelity models within a multiscale hierarchy, enabling dynamic, bidirectional feedback between macro- and microlayers. This integrated perspective employs an indispensable role in smart cities. For instance, neglecting cross-scale interaction may intensify traffic oscillations [11] and reduce control efficiency [12] in transportation subsystems. In communication subsystems, macrolevel states can be utilized to guide microlevel strategies [13], achieving coordinated, closed-loop optimization across scales. This review uniquely focuses on resolving this macro–micro mismatch through feedback modeling, rather than treating the 2 scales as disjoint analytical systems. Systemic incoherence arises when models at different scales yield contradictory predictions. For example, in transportation systems, macroscopic traffic flow models based on fluid dynamics analogies, such as the Lighthill–Whitham–Richards equations, effectively predict congestion waves yet fail to account for the microscopic heterogeneity of driver decision-making during accidents or route diversions [14]. The persistent divide between macro- and microscale analyses engenders 3 fundamental issues in developing smart cities, comprising theoretical incompleteness, falsifiability vacuumization, and causal emergence blur:

• Theoretical incompleteness. Macroscopic studies idealize cities as uniform entities. These assumptions create logical contradictions when explaining emergent anomalies. Conversely, microscopic investigations prioritize individual behavior but fail to reveal how such behaviors coalesce into city-level patterns.
• Falsifiability vacuumization. Macromodels rely on idealized parameters that lack empirical grounding, reducing theories to speculative formalisms. Meanwhile, microresearch drowns in stochastic noise (e.g., random deviations in individual decisions), rendering models computationally intractable. Both approaches create unfalsifiable systems, meaning that discrepancies can be perpetually “fixed” by supplementing new parameters or data granularity, eroding scientific accountability.
• Causal emergence blur. Macroperspectives attribute urban dynamics to top-down laws (e.g., population growth), erasing pivotal local triggers (e.g., a metro station sparking regional transformation). Micromodels detect granular events but cannot distinguish isolated incidents from systemic risks. This fragmentation blinds researchers to cross-scale causation; i.e., critical “butterfly effects” between scales are lost, while localized noise masquerades as meaningful signals, crippling predictive and intervention capacities [15].

Urban mobility systems are neither fully deterministic machines nor purely chaotic aggregates. As a typical complex megasystem, their operational logic is intertwined with the dynamic coupling of microlevel individual behaviors and macrolevel structural evolution, exhibiting prominent characteristics such as openness, nonlinearity, hierarchical nesting, and emergence. Dual-scale blindness mirrors trying to map a fractal with Euclidean geometry, losing resolution when zooming in, and overlooking systemic patterns when zooming out. Breaking the limitations of single-scale analysis and constructing organic connections between macrolevel laws and microlevel mechanisms through iterative feedback, enabling disturbances at one scale to recalibrate behaviors at the other scale, has become key to solving the challenges of urban mobility. In the context of artificial intelligence (AI), a resolution lies in the macro–micro integration that simultaneously encodes large-scale stability and amplifies microscale “tipping-point” dynamics through cascading feedback across transportation, communication, and energy networks that comprise the mega-mobility system in smart cities [16]. In this review, we undertake a comprehensive discussion of the MMIF paradigm empowered by AI for analyzing mega-mobility systems in smart cities. We systematically elaborate on the essential characteristics and modeling logic of typical constituent subsystems and provide an interdisciplinary perspective as well as a viable methodological protocol for investigating other complex multinetwork systems.

The remainder of this paper is organized as follows: Nature of Mega-mobility Systems in Cities introduces the MMIF paradigm for analyzing mega-mobility systems and delineates its essential attributes through the perspective of complexity science. Against the backdrop of AI-powered urban intelligence advancements, Cutting-Edge AI Methods and Technologies in MMIF for Analyzing Mega-mobility Systems investigates the cutting-edge methods and technologies that contribute to the MMIF paradigm. Typical Constituent Subsystems and the Role of MMIF further presents a systematic deconstruction and analysis of typical constituent subsystems. Challenges and Perspectives discusses the remaining challenges and future opportunities, while Conclusions draws conclusions.

## Nature of Mega-mobility Systems in Cities

**Fig. 2.**
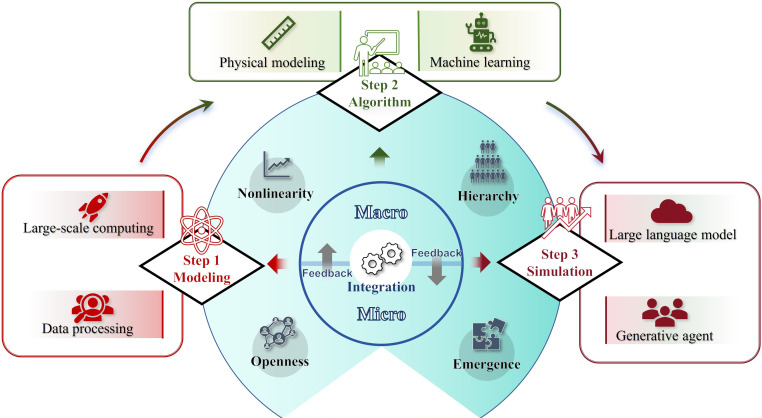
Macro–micro integration with feedback (MMIF) and its integral cutting-edge artificial intelligence (AI) methods and technologies.

To effectively understand and manage urban environments and mobility systems, MMIF has become essential in addressing their multifaceted nature. Macro–micro integration facilitates advancements across 3 interrelated aspects: modeling, which abstracts and represents system dynamics across scales; algorithms, which process and optimize interactions among diverse components; and simulation, which provides testable, reproducible environments for validating urban policies and human behaviors. In practice, the operation of MMIF follows a cyclic workflow that logically connects its components. First, modeling provides structural abstractions at different scales: macrolevel models describe citywide patterns, and microlevel formulations capture the detailed behaviors of individual agents. Second, algorithms act as computational drivers, ranging from global optimization at the macroscale, to decision rules at the microscale. Third, simulation integrates these elements into testable environments, enabling policy scenarios to be examined under reproducible conditions. Finally, feedback loops transmit information bidirectionally, whereby macrolevel system states influence local behaviors, while microlevel variations accumulate upward to reshape overall system dynamics. This iterative process—modeling, algorithm design, simulation, and cross-scale feedback—constitutes the operational logic of MMIF, ensuring that urban mobility systems can be studied and managed in a coherent manner. As shown in Fig. 2, these components are not isolated but are logically connected through feedback loops and directional interactions, collectively forming the foundation for exploring and intervening in smart cities.

Urban mobility systems are not merely agglomerations of components; they are characterized by 4 features that define their behaviors and structures, i.e., openness, nonlinearity, hierarchy, and emergence. These features interact dynamically across macro- and microscales, shaping both the stability and adaptability of urban environments. The interplay of these characteristics underlies the need for MMIF in both theoretical studies and real-world applications.

### Openness

The openness of urban mobility systems refers to the continuous exchange of matter, energy, information, and resources between the system and its external environment. As one of the most essential characteristics, openness enables systems to continuously adapt to environmental changes, thereby maintaining dynamic equilibrium and evolutionary capacity. In MMIF, this feature is reflected in how microscopic agents respond to macroscopic environmental conditions. For instance, individual vehicle behaviors (e.g., speed adjustments) are influenced by macroscopic traffic conditions, while these microlevel decisions collectively shape emergent traffic flow patterns [9]. This interplay underscores the importance of integrating macrolevel insights with microlevel actions in the MMIF framework, allowing for a more comprehensive understanding of how urban mobility systems adapt and evolve in response to external stimuli. By leveraging AI-driven analyses, MMIF can enhance the responsiveness of urban mobility systems, ensuring that they remain resilient and efficient in the face of changing conditions.

### Nonlinearity

Nonlinear systems are those that do not satisfy the principle of superposition, meaning that the system’s output changes are not proportional to its input changes. In other words, the whole does not equal the algebraic sum of its parts. Nonlinearity represents one of the most fundamental characteristics of urban mobility systems and thus is naturally embedded in MMIF, where minute changes in initial conditions may trigger substantial changes or abrupt transitions in macroscopic system behaviors. For example, minor disruptions like sudden braking can propagate in a nonlinear fashion, creating widespread traffic congestion, and localized panic reactions in a crowd can rapidly escalate into chaotic stampedes [17]. From a computational perspective, nonlinearity often increases the difficulty of solving large-scale problems, as analytical tractability is precluded and algorithmic complexity is amplified. While some structured nonlinearities can be reformulated into linear equivalents, many real-world nonlinear interactions—such as agent interactions with feedbacks or chaotic dynamics—remain fundamentally hard to approximate or scale. By integrating both macrolevel patterns and microlevel interactions, MMIF can effectively capture the inherent nonlinearity of urban mobility systems, enabling more accurate predictions and interventions that account for the complex interdependencies between individual behaviors and collective outcomes.

### Hierarchy

The hierarchical organization of urban mobility systems involves the composition of multiple interrelated subsystems distributed across different levels; each subsystem maintains distinct structural and functional properties while contributing to higher-order system behaviors. This architectural layering enables functional integration and coordination across scales, which is key to MMIF, thus enhancing both the stability and adaptability of the overall system. Through a feedback loop between levels, lower-level entities (e.g., individual vehicles) interact to give rise to macrolevel configurations (e.g., network flows), while, in turn, higher levels impose top-down constraints that regulate microlevel dynamics [9]. In MMIF, this hierarchical structure is essential for facilitating coordinated control between macro- and microscales, allowing for the dynamic adjustment of strategies based on real-time feedback.

### Emergence

Emergence refers to the rise of novel system-level behaviors or properties that are neither present in, nor predictable from, the characteristics of individual components. It typically results from the nonlinear interactions, feedback loops, and self-organizing processes distributed across the system. At the core of this concept lies the notion, which underpins MMIF, that the collective dynamics of microscopic elements can generate coherent macrolevel patterns. Studies on agent-based simulation have demonstrated that aggregated mobility patterns in urban areas can emerge from decentralized decision-making processes by individual drivers [18]; this phenomenon cannot be fully understood by analyzing each driver and exemplifies the emergence of global complexity from local interactions. In other words, the macrolevel regularities are not imposed externally but instead arise organically from within the system, reinforcing emergence as a fundamental mechanism in MMIF. By effectively integrating macro- and microperspectives, MMIF captures the essence of emergent behaviors, allowing for a deeper understanding of how local interactions can lead to coherent macro-impacts, such as traffic congestion or efficient public transportation usage.

## Cutting-Edge AI Methods and Technologies in MMIF for Analyzing Mega-mobility Systems

In the context of openness, nonlinearity, hierarchy, and emergence of urban mobility systems, recent methodological and technological advancements in AI have largely facilitated the MMIF realization to meet the modeling, computation, optimization, and control needs posed by the smart city initiative. These cutting-edge AI methods and technologies, featuring heterogeneous data fusion [19], large-scale intelligent computing [20], knowledge–data co-driven modeling [21], and generative large language models (LLMs) [22], have revolutionized our ways of understanding and analyzing urban mobility systems. They are tailored to exploit the interdependence of different scales, enabling adaptive responses, real-time forecasts, and emergent pattern discoveries. As a result, they serve as crucial tools for bridging microlevel decisions (e.g., individual mobility demand and choices) with macrolevel outcomes (e.g., congestion patterns and infrastructure resilience).

### Multisource heterogeneous data acquisition and fusion

The openness of mega-mobility systems demands a technological framework capable of integrating multidimensional data streams to bridge the gap between microlevel behaviors and macrolevel phenomena. MMIF clearly illustrates the closed loop between microlevel actions and macrolevel responses. Traditional single-source modeling approaches often fail to capture the interactions between individuals and groups, resulting in marked deviations between simulated outcomes and the actual dynamic evolution of the system [23]. Such limitations manifest in urban challenges like traffic congestion, energy fluctuations, and information silos. By positioning MMIF as the core, data ingestion, model inference, and control decisions are ensured to operate within a continuous feedback loop. The integration of multisource heterogeneous data-spanning physical sensors, social platforms, traffic signals, and environmental monitors serves not only to expand the scope of perception but also to construct a semantically aligned representation of the open urban system. These efforts constitute the data layer of MMIF, enabling real-time capture of both macrodynamics and microbehaviors. By establishing interconnectivity across previously siloed transportation subsystems, these technologies offer the foundational scaffolding for open-system modeling and macro–micro integration.

Multisource heterogeneous data fusion technologies provide a robust data foundation for MMIF modeling by constructing cross-spatiotemporal-scale correlation networks [24]. This framework incorporates multimodal data sources, including physical sensors (e.g., smart meters and roadside detectors), virtual sensing (e.g., mobile signaling and social media footprints), and environmental parameters (e.g., 3-dimensional terrain models and real-time meteorological data), forming a comprehensive perceptual architecture. To overcome the challenges of integrating heterogeneous data, state-of-the-art research employs multi-level processing techniques. In the preprocessing stage, adaptive filtering algorithms [25], spatiotemporal interpolation [26], and knowledge distillation methods [27] are used for noise reduction, missing data imputation, and resolution alignment. At the feature fusion level, techniques such as dynamic graph neural networks [28], tensor decomposition [19], and knowledge graphs [29] are employed for dimensionality reduction and unified feature representation. These enable the coupling analysis of discrete transportation patterns, energy networks, and social behavior dynamics. These methods feed into MMIF, where model outputs are continuously compared with the observed microbehavior to improve subsequent predictions. The core advantage of this technology lies in its multisystem cross-validation mechanism, which enhances modeling completeness and decision robustness. For instance, spatiotemporal consistency analysis corrects systemic biases in single data sources, while cross-domain validation reveals cascading the propagation pathways of complex events. Finally, MMIF leverages this validated knowledge to adaptively optimize urban mobility strategies in real time. These methods not only improve the accuracy of MMIF modeling but also establish a theoretical foundation for resilience optimization and adaptive control in urban mobility systems, embodying the technological evolution logic of “holistic perception–global deduction–global coordination” in open systems. A concrete example of the application of multisource data fusion in urban traffic is a spatiotemporal method [30], which fuses real-time Global Positioning System (GPS) trajectories, loop detector data, and environmental parameters. The data-driven framework achieved a 15-min prediction horizon for citywide congestion events with over 92% accuracy, notably outperforming single-source models. Notably, it enabled proactive traffic management interventions, reducing average delay times by 18% during peak hours in a metropolitan test bed.

### Large-scale intelligent computing

The nonlinear dynamics of urban mobility systems (e.g., stochastic traveler behavior, cascading phase transitions in energy networks, and chaotic information diffusion) pose 2 computational challenges: precise resolution of microlevel heterogeneous interactions and extraction of macrolevel patterns from ultralarge-scale systems. This section situates large-scale intelligent computing within the MMIF framework, emphasizing how MMIF leverages high-performance architectures to realize continuous macro–micro inference and feedback refinement. Traditional models, constrained by the balance between computational efficiency and accuracy, often resort to quasi-static assumptions or mean-field approximations, leading to microlevel distortion and macrolevel resolution loss. To faithfully simulate nonlinear interdependencies, the deployment of large-scale intelligent computing frameworks enables dynamic, real-time inference of multi-agent behaviors and system-level emergent phenomena. These technologies directly correspond to the nonlinear characteristic by facilitating the accurate capture of feedback loops, stochastic perturbations, and nonequilibrium dynamics.

Recent advances in intelligent computing address these challenges through synergistic innovations in heterogeneous parallel architectures [31]. Spatial decomposition strategies partition urban systems into physically meaningful submodules [20] (e.g., traffic network zones and virtual power grid units), enabling distributed and parallel computing via the message passing interface. Graphics processing unit (GPU)-accelerated matrix operations optimize tensor computations, reducing the complexity of agent-based simulations from exponential to near-linear scales by transforming micro-interaction rules into parallelizable matrix operations [32]. This spatial-partitioning and data-parallel architecture forms the computational backbone of MMIF, alleviating the double curse of dimensions in macro–micro modeling. Additionally, cloud-edge collaborative computing further extends computational boundaries through MMIF dynamic resource allocation, balancing workloads between edge nodes and cloud supercomputers to enable real-time state inference under communication latency constraints. For instance, the efficacy of large-scale intelligent computing is evidenced by a recent study focusing on urban traffic flow simulation. Researchers developed a GPU-accelerated framework based on the Regional Scale Traffic Simulation Framework to parallelize an agent-based model of vehicle movements across a city-scale network [32]. By transforming micro-interaction rules into parallelizable matrix operations, the simulation complexity was reduced from an exponential to a near-linear scale. Experimental results demonstrate that the intelligent computing framework is capable of completing simulations of 2.82 million trips in just 6.28 min on a single GPU machine equipped with 5,120 CUDA cores (Tesla V100-SXM2).

### Knowledge–data co-driven modeling

The multilayered nature of urban mega-mobility system calls for the MMIF modeling framework, which achieves both physical interpretability and data-driven adaptability. Traditional single-paradigm approaches face a fundamental trade-off: purely mechanistic models oversimplify system heterogeneity, while purely data-driven models lack credibility in predicting critical phase transitions due to their black-box nature [21]. To reconcile this trade-off, hybrid modeling strategies offer a structurally coherent way to reflect system hierarchy. By explicitly aligning macrolevel laws with microlevel flexibility, these methods naturally preserve the multi-level interdependencies among transportation subsystems.

Current breakthroughs in MMIF focus on multimodal fusion mechanisms. One approach embeds conservation laws and constitutive equations as structural constraints in machine learning models, e.g., incorporating hard physical constraints in neural networks or soft conservation constraints in loss functions [33]. Another strategy constructs a bi-level framework, where neural networks predict macrotrends, while physics-based solvers model micro-interactions [34]. This “white-box–black-box” architecture enhances cross-scale consistency, enabling synergistic prediction of microtrajectories (e.g., vehicle movements) and macrophenomena (e.g., traffic congestion). For instance, a physics-informed deep learning framework was developed that combined second-order traffic flow models and neural networks to solve the traffic state estimation problem [35]. When applied to a large urban network, this hybrid model achieved a higher accuracy in reconstructing and predicting traffic densities under low-data observability conditions compared to a purely data-driven baseline while also ensuring that predictions adhered to fundamental traffic flow principles.

### Generative agents and causal reasoning by LLMs

The dynamic evolution of urban transportation systems inherently embodies a dialectical unification of an MMIF process between microscopic individual interactions and macroscopic patterns. For instance, generative agents employ deep self-attention mechanisms to model the behavioral patterns of massive microscopic entities (e.g., vehicles and pedestrians), thereby uncovering implicit correlations between individual decisions and collective order, known as the phenomenon of emergence in MMIF [36]. Within this context, the paradigm of coarse-grained governance has emerged: rather than pursuing precise control over every microscopic entity, it extracts the statistical regularities of group behaviors. When governance granularity aligns with the system’s intrinsic correlation length, information loss at the microlevel does not reduce efficiency but enhances decision robustness by filtering noise and closing MMIF loops in self-organizing critical states.

MMIF further combines generative models and causal reasoning technologies within a novel methodological framework. This is particularly valuable in emergency management scenarios, where high uncertainty and sparse historical data necessitate the inference of emergent patterns and latent decision logic, thereby enhancing system robustness and adaptability. Confronted with incidents such as accidents or extreme weather events, conventional models lack analytical capabilities. Transformer-based generative models extract the latent variables of individual mobility patterns (e.g., risk aversion coefficients and route-switching thresholds) through self-attention mechanisms and construct emergent dynamics equations for multi-agent interactions. This generation–emergence bidirectional mapping mechanism enables regional traffic capacity metrics to dynamically reflect cumulative microscopic behaviors. Cutting-edge research is integrating counterfactual reasoning into urban mega-mobility system analysis frameworks. Causal discovery algorithms powered by LLMs facilitate vulnerability node prediction through comparisons between real-world states and intervention-based counterfactual scenarios. Such capabilities empower decision-makers to preemptively identify critical infrastructure weaknesses. Explainable AI technologies further reveal microscopic parameter sensitivity mappings, thereby providing quantitative foundations for resilient urban design. This MMIF paradigm shift transforms complexity science from passive observation into active emergence design. Future systems will not only predict critical phenomena but also induce the desired macroscopic order through proactive information granularity modulation, forging a pathway for urban emergency response in extreme scenarios.

## Typical Constituent Subsystems and the Role of MMIF

Over the past decade, macro–micro integration has systematically incorporated cutting-edge insights from complexity science, computer science, and cyberphysical systems, offering actionable solutions. This section investigates the paradigm of macro–micro integration within 3 critical domains of subsystems: urban transportation, communication, and energy systems (see Table 1).

**Table 1.** Typical constituent subsystems of an urban mega-mobility system

Subsystems	Components	Macrolevel focus	Microlevel objectives	Typical applications
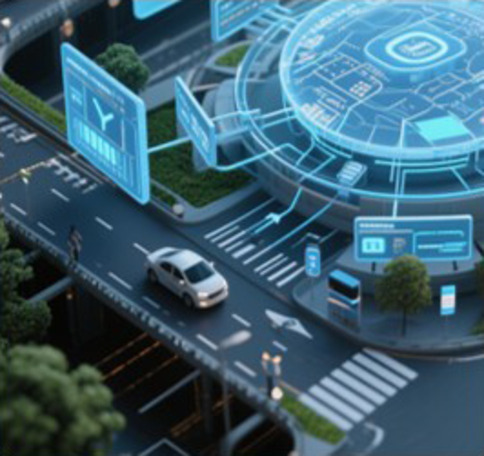 Urban transportation subsystem	Multimodal transportation	• Urban travel structure • Real-time congestion	• Individual travel chain • Road section capacity	• Traffic flow prediction • Public traffic network optimization
	Logistics and supply chains	• Demand forecasting • Resource allocation	• Route optimization • Last-mile scheduling	• Last-mile delivery optimization • Emergency network design
	Intelligent control infrastructure	• Traffic state prediction • Regional coordination	• Traffic safety • Signal control	• Parking behavior simulation • Adaptive signal optimization
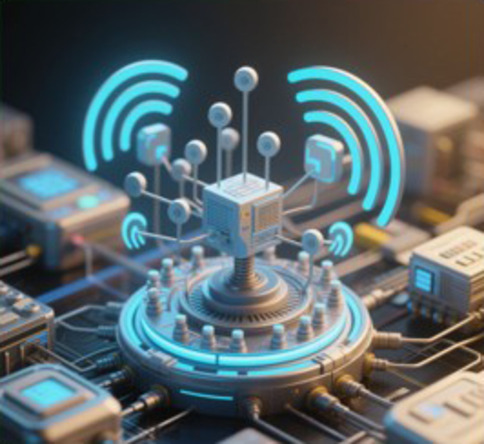 Urban communication subsystem	Wireless	• Network topology • Spectrum utilization	• Resource allocation • Device performance	• 5G cellular network • V2X
	Core network	• Network coverage • Spectrum capacity	• Channel characteristics • Interference suppression	• Internet of Things • Edge computing
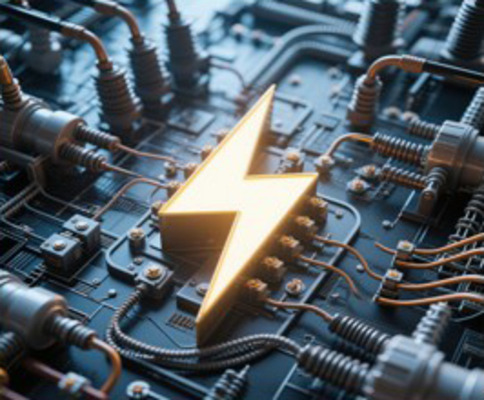 Urban energy subsystem	Generation and transmission	• Facility spatial layout • Power grid capacity planning	• Charging behavior • Implementation of V2G function	• Demand forecasting of the OD matrix • Dynamic electricity price guidance
	Distribution and consumption	• Peak-shaving and load-shifting strategies • Transportation–energy coupling optimization	• SOC real-time feedback • Bidirectional V2X power exchange	• Optimal configuration of charging stations • Bidirectional feedback system

5G, fifth generation; V2X, vehicle-to-everything; V2G, vehicle-to-grid; OD, origin–destination; SOC, state of charge

### Mobility-oriented urban transportation subsystem

**Fig. 3.**
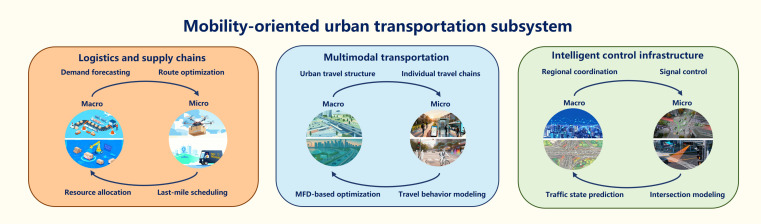
Analysis of the transportation subsystem in the context of MMIF. MFD, macroscopic fundamental diagram.

The urban transportation system plays important roles in the operation of an urban mega-mobility system, which undertakes the flow of goods and the commuting of residents. As a typical complex system, the urban transportation subsystem includes 3 components: multimodal transportation, logistics and supply chains, and intelligent control infrastructure. The multimodal transportation system meets the travel needs of residents, the logistics and supply chain system support the material metabolism cycle, and the intelligent control infrastructure forms a digital control center (see Fig. 3). The complexity of a complex transportation system reflects its cross-scale butterfly effect [37], indicating the necessity of coupling macro- and microlevels for research.

• Multimodal transportation. Multimodal transportation combines public transit, private vehicles, shared mobility, and pedestrian travel, forming the core infrastructure of urban transport networks. Key challenges that multimodal transportation faces include difficulties in integrating data across transportation modes, insufficient infrastructure compatibility, and the low attractiveness of public transportation.
  From the perspective of macroscopic objectives, due to the deepening adoption of transit-oriented development concepts and sustainable principles, optimizing and coordinating public transportation is a primary goal. The macroscopic fundamental diagram serves as a core tool, extended to bimodal systems to analyze the cumulative impacts of competing transportation modes on network capacity. Existing studies also utilize a bimodal network to analyze the velocity distribution and traffic flow characteristics of different modes of transportation [38]. At the microscopic level, the individual travel chain is critical. Data sources for these chains include travel surveys, GPS data, automated fare collection systems, and mobile signaling data. Analytical methods include statistical regression models [39], discrete choice models [40], structural equations [41], and machine learning [42].
• Logistics and supply chains. Logistics and supply chains integrate logistics resources and supply chain management technologies to achieve full-chain coordination from raw material procurement, production, and warehousing to last-mile delivery. The main challenges it faces are multiobjective, cross-scale optimization of logistics networks, requiring balanced consideration of supply–demand equilibrium, the network structure, and route allocation. In addition, the connection with multimodal transportation is also a key factor as the transportation time and cost depend on traffic congestion and stochasticity.
  At the macroscopic level, understanding the dynamic relationship between logistics demand and economic factors is critical. System dynamics models [43], origin–destination–service models [44], and machine learning [45] are widely used. Urban logistics network optimization includes structural optimization, resource allocation, and facility distribution. Methods like complex network theory [46] and mixed-integer linear programming models [47] are commonly applied. From the perspective of microscopic objectives, research addresses transport route optimization and multimodal logistics scheduling. Last-mile delivery is a key challenge, with crowdsourced logistics offering solutions. Some studies proposed routing algorithms for assigning delivery tasks to temporary drivers (such as commuters in the city). The rise of the low-altitude economy also provides new ideas for terminal distribution problems.
• Intelligent control infrastructure. Intelligent control infrastructure is a multi-level, multimodule integrated framework, focusing on multidimensional collaborative optimization and dynamic regulation. The intelligent control infrastructure emphasizes signal control optimization, traffic flow dynamics regulation, urban parking management, and guidance of complex traffic networks to achieve the purpose of alleviating traffic congestion, constructing a global perception–decision–control closed loop.
  At the macroscopic scale, research focuses on macroscopic regional coordination and boundary control. Hierarchical control strategies combine upper-layer regional boundary flow regulation with lower-layer local signal optimization [48]. Deep learning [49] and reinforcement learning [50] are applied for traffic state modeling, prediction and decision optimization. Deep reinforcement learning provides generalization abilities for diverse traffic scenarios. From the perspective of microscopic objectives, research addresses vehicle behavior simulation and signal control optimization. Beyond traditional transportation modes, autonomous vehicle technology is a growing focus [51]. Investigations of road environment impacts on autonomous vehicle safety and interactions between autonomous vehicles and human-driven vehicles aim to enhance mixed traffic safety and efficiency.

#### MMIF for the mobility-oriented urban transportation subsystem

Single-scale analysis risks an incomplete understanding of urban mobility systems, exemplified by overlooking microscopic individual behavior on macroscopic traffic patterns. Parameter inconsistencies across scales may induce falsifiability vacuums, while neglecting multiscale interactions obscures cross-level causation (e.g., causal emergence ambiguities).

A multi-level collaborative framework is urgently needed to couple macroscopic and microscopic layers. Macro–micro integration of multimodal traffic aims to integrate macroscopic traffic dynamics and microscopic behavioral models. Key research areas include parameter consistency [52], multidimensional travel behavior modeling [53], and traffic system simulation [54]. With the continuous iteration and evolution of generative AI, LLMs can better adapt to massive traffic flow data, capture spatial–temporal correlation, and provide solutions for MMIF modeling in multimodal traffic systems through multimodal data fusion, generative simulation, and cross-scale attention mechanism. In the research of logistics and supply chains, isolated macroscopic or microscopic studies lead to imbalanced resource allocation and inefficiency. MMIF emphasizes optimizing system-wide efficiency and resource allocation by linking global system behavior with individual actions. Beyond technical efficiency, MMIF should aim to create social outcomes such as reduced travel times, improved safety, and ensuring fair access to transportation services, especially for marginalized communities [55,56]. As for intelligent control infrastructure, focusing solely on the macroscopic level exacerbates traffic oscillations [11], reduces control strategy efficiency [12], and increases safety risks in mixed traffic between autonomous vehicles and human-driven vehicles [57]. Thus, integrating both levels is critical.

With the MMIF paradigm, the transportation subsystem exhibits bidirectional dependencies on both communication and energy subsystems. Communication subsystems supply real-time data for dynamic routing and congestion mitigation, while travel patterns shape network load and coverage requirements. Similarly, transportation electrification introduces spatiotemporally varying charging demands on the grid, whereas energy availability and pricing directly influence travel behavior and mode selection.

### Mobility-oriented urban communication subsystem

**Fig. 4.**
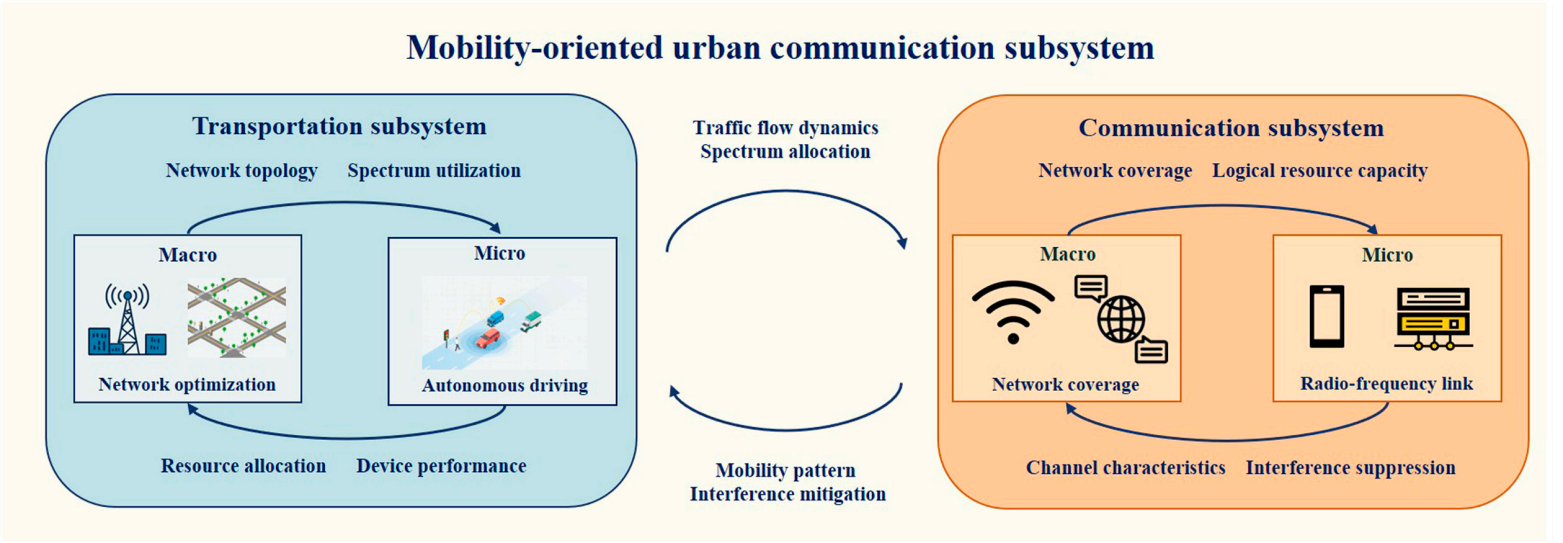
Analysis of the mobility-oriented urban communication subsystem.

As a core subsystem of the urban mega-mobility system, the mobility-oriented urban communication subsystem integrates road traffic networks with modern communication technologies, including wireless systems (fourth-generation/fifth-generation cellular networks and vehicle-to-everything [V2X]) and core network systems (the Internet of Things and edge computing), thereby forming the backbone of mobility intelligence. Its design and operation should incorporate both macrolevel architecture and microlevel technical details to ensure efficiency and stability [58]. Based on this, MMIF bridges the gap between macrometrics and microperformance, enabling precise prediction, optimized design, and enhanced system performance and adaptability (see Fig. 4).

Given the time-varying characteristics and dynamic network topology of the transportation subsystem and communication subsystem (wireless system and core network system), mobility intelligence requires an MMIF paradigm that synchronizes vehicle and base station information, balances user flows, and mitigates environmental interference. Accurately spatial–temporal paradigm of network topology enables efficient resource allocation, enhances stability, and improves overall communication efficiency [59]. At the macrolevel, optimization focuses on network coverage, spectrum allocation, and capacity. For example, high-speed vehicles and frequent handovers demand adaptive base station placement considering interference-aware spectrum management to maximize spectrum efficiency and minimize service disruptions. Therefore, macrolevel optimization must jointly address deployment, load balancing, and dynamic spectrum utilization. At the microlevel, research targets signal propagation characteristics [60], radio-frequency device performance [61], and protocol layer mechanisms [62] within the wireless system. By fine-tuning parameters such as signal-to-noise ratio, bandwidth allocation, and transmit power, interference is reduced and throughput is improved. Together, optimization at both the macro- and microlevels forms a synergistic framework: macrostrategies (network layout, core system architecture, and spectrum management) establish the interference landscape, while microlevel adjustments and feedback enhance capacity and coverage.

#### MMIF for the mobility-oriented urban communication subsystem

The mobility-oriented urban communication subsystem is a complex engineering system, which is composed of billions of equipment terminal devices (such as onboard units and roadside units) interconnected through heterogeneous networks (cellular networks and V2X communication). Its topology presents the dual characteristics of a scale-free and small-world network: the degree distribution follows a power law, and the average path length increases only slowly as the network scales. Single-scale communication modeling methods face 3 important challenges arising from theoretical incompleteness, falsifiability vacuumization, and causal emergence blur. Theoretical incompleteness arises when microlevel models (such as the Rayleigh fading model) fail to capture the network-level quality-of-service degradation caused by a small number of anomalous devices; falsifiability vacuumization is due to the fact that statistical methods, while adept at characterizing channel behavior through large amounts of measurement data, lose their applicability in new environments due to their reliance on specific datasets; and causal emergence blur occurs when local events (such as city-level base station failures) are misinterpreted as global algorithm failures, or when microlevel optimization measures such as terminal power-saving modes trigger macrolevel instability, thereby blurring clear causal relationships. Given such structural complexity, there is an urgent need for MMIF models.

Mobility intelligence employs an MMIF paradigm. Specifically, it guides microlevel strategies (e.g., V2X channel allocation and roadside unit power control) based on macrolevel road network states (e.g., regional traffic flow and communication spectrum utilization) [13]. For instance, by coupling the macroscopic fundamental diagram with the communication bandwidth in the core network system, the system can forecast congested zones and adjust signal timing and V2X resource scheduling in advance. At the microlevel, vehicles interact with multisource sensing and communication units to exchange local state information (position and speed) with nearby edge servers. These updates drive iterative refinements of the MMIF paradigm, ensuring that local traffic dynamics are captured in detail while still optimizing overall network performance. Moreover, communication subsystems must also reflect societal factors, such as user trust in the system and the social impact of communication disruptions or delays, which may have a cascading effect on public perception and system adoption [63,64]. In recent years, the MMIF paradigm has increasingly leveraged heterogeneous data fusion and large-scale intelligent computing to achieve real-time perception and coordinated control in complex urban environments. This computing framework tightly combines core-network system, cloud, and edge resources in a distributed agent architecture. The cloud leverages its centralized processing and storage capabilities to generate city flow forecasts by integrating multisource data, while edge nodes carry out low-latency computations to minimize communication overhead and facilitate microlevel control [65].

By combining these elements, the MMIF paradigm captures local dynamics and ensures efficient, scalable coordination across the urban mega-mobility and communication system. The communication subsystem operates as the neural layer connecting transportation and energy systems. It enables real-time data exchange for traffic management, coordinates energy distribution and electric vehicle (EV) charging schedules, and provides feedback channels that synchronize vehicle flow with power demand. By modeling these dependencies jointly, the MMIF paradigm supports scalable and adaptive cross-domain coordination.

### Mobility-oriented urban energy subsystem

**Fig. 5.**
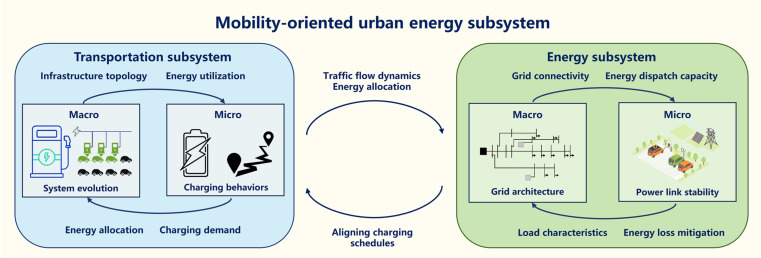
Analysis of the energy subsystem in the context of MMIF.

The urban energy subsystem is a key subsystem for building intelligent, green, and resilient cities within an urban mega-mobility system. It breaks the traditional boundaries of independent operation between urban transportation system and energy system, achieving overall optimization through bidirectional interaction (see Fig. 5). Based on the MMIF framework, urban transportation–energy integration represents a sustainable synergy that goes beyond simple combination, achieving deep integration through bidirectional coupling that enhances both environmental sustainability and system efficiency. Current research mainly focuses on various individual aspects of the coupling between urban transportation and energy subsystems, including the estimation of charging demand [66] and the impact of the urban mobility system on the energy network [67].

In the mobility-oriented urban energy subsystem, the road network–energy network synergy has established a bidirectional real-time feedback loop for traffic behavior decision-making and energy network responses, addressing the decoupling dilemma of “travel–energy supply” through multi-level data fusion. At the macrolevel, the focus of the research is on system evolution and impact assessment, minimizing traffic delays and energy losses [68]. For instance, it predicts the spatiotemporal distribution of EV charging demand at the city scale based on big data related to travel (origin–destination matrix and travel chains) [69]. Simultaneously, it optimizes the spatial layout, capacity planning of large-scale EV charging infrastructure (fast-charging stations, battery-swapping stations, and slow-charging piles), and the expansion and upgrading of the power grid in a coordinated manner. Meanwhile, the research at the microlevel emphasizes guiding and modeling individual behaviors, including travel behavior [70] and charging behavior [71].

#### MMIF for the mobility-oriented urban energy subsystem

The MMIF framework facilitates sustainable synergy by creating feedback loops that simultaneously optimize mobility efficiency and energy sustainability, demonstrating how integrated planning can achieve environmental and economic co-benefits. The mobility-oriented urban energy subsystem exemplifies sustainable synergy through its multi-level integration approach, where transportation and energy systems coevolve to create solutions that are environmentally responsible, economically viable, and socially beneficial. The complexity of the integrated urban transportation and energy subsystem arises from its dynamic coupling. It manifests as a multi-level nested structure: the microlevel dynamics of vehicle battery charging and discharging emerge as macrolevel load peak and valley characteristics through aggregation effects [72], while macrolevel power grid frequency fluctuations or electricity price signals, in turn, constrain microlevel charging behavior choices [73]. Single-level investigations face substantial limitations when confronted with this complexity.

The core mechanism of MMIF for mobility-oriented urban energy subsystem lies in establishing a bidirectional real-time feedback loop between travel behavior and energy network response. This approach leverages multilayer data fusion to address the decoupling dilemma of travel–energy supply. In the top-down pathway, based on predicted macro-charging demand, rapid-charging station prices are dynamically adjusted to induce individual micro-path choices that avoid high-price congestion areas [74]. In the bottom-up pathway, real-time data feedback from individual vehicle battery state of charge is incorporated into the macromodel to calibrate spatiotemporal charging demand and congestion propagation paths [75]. In recent years, macro–micro integration has been widely applied to vehicle–grid integration [76]. Vehicle–grid integration is not only about connecting EVs to the grid but also about combining macrolevel electric power demand management with microlevel individual vehicle charging and discharging behaviors to achieve efficient energy utilization and optimized scheduling. At the macrolevel, the grid can dynamically adjust electricity prices based on real-time load and renewable energy generation, encouraging users to charge during off-peak hours to balance electricity supply and demand; at the microlevel, vehicle owners can use smart charging systems to choose the best charging time and method and even feed energy back to the grid from their vehicle’s battery during peak times, creating a bidirectional flow of energy. Additionally, human charging behavior is heterogeneous, driven by range anxiety, price sensitivity, and daily routines, leading to stochastic grid load fluctuations [77]. Social dynamics influence EV adoption and charging behavior, while demand–response participation depends on convenience, incentives, and environmental awareness [78].

This macro–micro integration modeling not only enhances the flexibility and stability of the grid but also creates economic benefits for EV users, driving the development of sustainable urban mega-mobility. The energy subsystem forms the power foundation for both transportation electrification and communication infrastructure operations. It must dynamically adapt to transportation-driven charging demands while ensuring stable power supply for communication networks. Conversely, predictive grid management relies on communication networks for coordination and leverages transportation patterns for demand forecasting, demonstrating the deep interdependence among all 3 subsystems in achieving sustainable urban development.

## Challenges and Perspectives

Building upon the demonstrated success of MMIF in urban mega-mobility systems, it is evident that this paradigm has proven instrumental in decoding nonlinear dynamics, cross-scale feedback, and emergent behaviors in smart cities. Through concrete implementations ranging from data-driven modeling of traffic congestion to cascading failure prediction in power grids, MMIF has unlocked new levels of analytical depth and system-wide optimization.

However, the practical realization of these advancements across domains reveals a shared set of scientific, technological, and ethical challenges. These challenges arise from efforts to ensure the reliability of models that simulate emergent and nonlinear urban phenomena, deploy scalable and interoperable computing infrastructures under real-time constraints, and safeguard the social fairness and transparency of automated decisions. The following sections explore these challenges while outlining future opportunities to extend the MMIF toward more robust, adaptable, and ethically grounded urban intelligence.

### Scientific challenges

As MMIF transitions from experimental tools to operational engines for urban mega-mobility systems, the scope of their objectives expands: from modeling complexity to enabling anticipation and supporting transparent, system-wide interventions. Realizing this vision demands addressing the current limitations in adaptability, generalization, and interpretability that constrain MMIF in dynamic, high-dimensional urban environments. These challenges are most evident in 3 areas: the verification of complex system behavior, the granularity of mesoscopic modeling, and the consistency of macro–micro model coupling.

• Verification and interpretability of complex urban systems. Due to the inherent nonlinearity, emergence, and dynamic nature of urban mega-mobility systems, MMIF encounters substantial challenges in terms of validation and interpretability. MMIF modeling and simulation often rely on extensive assumptions and parameter settings, making it difficult to guarantee reproducibility and reliability in real-world systems [79]. Meanwhile, with the widespread application of data-driven methods such as AI and machine learning, the “black-box” nature of models is becoming increasingly prominent, limiting the transparency and credibility of decision-making [80].
• Granularity and mesoscopic modeling. Existing MMIF models often struggle with determining the appropriate granularity for different subsystems. While microscopic models focus on individual agents and macroscopic models capture aggregated trends, mesoscopic modeling remains underdeveloped. A major challenge is designing mesoscopic models that can effectively bridge the gap between macro- and microscales while maintaining computational feasibility. One approach is to incorporate agent-based modeling with statistical aggregation techniques, enabling a more seamless transition between scales [81]. Additionally, data-driven mesoscopic models can leverage clustering techniques and dimensionality reduction methods to extract representative patterns from large-scale agent simulations [82]. To avoid potential ambiguity, we briefly clarify the 3 analytical tiers relevant to mega-mobility systems. The macrolevel captures system-wide dynamics, such as city-scale traffic flow patterns, while the microlevel focuses on individual-agent behaviors, such as vehicle driving maneuvers. Between them, a mesolevel scale can be recognized, typically addressing corridor or district-scale interactions. As an illustrative case, in transportation, congestion analysis at the mesolevel can be modeled using the cell transmission model, which explicitly captures the propagation of queues and bottlenecks across corridors. This lies between the fundamental diagram at the macrolevel, which aggregates flow–density–speed relationships, and car-following models at the microlevel, which reproduce stop-and-go waves and driver interactions. Similar bridging logic can be extended to other infrastructures. Introducing the mesolevel offers conceptual completeness and indicates how the MMIF framework can be extended to incorporate intermediate interactions when required, thereby strengthening its adaptability to diverse urban contexts.
• Inconsistency in macro–micro model coupling. The differences in temporal, spatial, and granularity scales between macro- and micromodels often lead to coupling inconsistencies. While macromodels emphasize overall trends, micromodels capture individual behaviors, resulting in potential scale-mapping distortions and accuracy loss during coupling. For instance, in travel demand forecasting for transportation systems, macromodels focus on regional flow predictions, while micromodels simulate individual travel chains. The differences in spatial and temporal resolution between the 2 may lead to simulation biases [83]. To ensure simulation reliability, strict consistency requirements must be enforced between macro–micro simulation tools, primarily across 2 critical dimensions. Firstly, network information consistency necessitates preserving higher-fidelity information within low-resolution macroscopic networks. The core challenge lies in modifying conventional macroscopic models and algorithms to maintain topological completeness and attribute accuracy during scale transitions. Secondly, mathematical model consistency requires maintaining derivational equivalence between frameworks. Through rigorous analytical derivation, this ensures that the macrolevel impedance function models remain mathematically reconcilable with microlevel models, thereby guaranteeing theoretical coherence across modeling hierarchies [53].

### Technological bottlenecks

The technical implementation of MMIF across urban domains is impeded by infrastructure-level constraints that limit real-time performance, scalability, and interoperability. While recent progress in data fusion and intelligent computing has expanded the theoretical capabilities of MMIF, translating these advances into operational, real-time systems has proven far more difficult.

• Deployable systems for real-time use. While many state-of-the-art algorithms demonstrate superior performance in controlled simulation environments, their integration into real-time operational systems is often hindered by platform incompatibilities, code generalizability issues, and the lack of modular software architectures [84]. For instance, deep-reinforcement-learning-based algorithms require constant state updates and tight coupling with data streams in the decision-making phase, yet many existing transportation management platforms still lack the real-time responsiveness needed to support such control strategies.
• Robustness and runtime safety. Urban environments are inherently noisy, with unexpected incidents such as sensor failures, cyberattacks, or abnormal demand surges. However, most current models do not offer built-in fault tolerance, online error correction, or graceful degradation mechanisms [85]. Without rigorous mechanisms to ensure system resilience, such as redundancy-aware control, online anomaly detection, or adversarial robustness training, intelligent decision-making systems may fail silently or generate erratic outputs under stress, undermining stakeholder trust.
• Cross-system data standardization. An urban mega-mobility system often involves multisource data from transportation, energy, communication, and other domains. Although multisource data fusion methods are increasingly sophisticated, their real-world implementation is often fragmented across agencies and vendors with incompatible data schemas, access controls, or update frequencies. Differences in format, timestamps, and spatial resolution hinder effective data fusion. Data fragmentation and inconsistency limit the accuracy and generalization ability of coupled models. This results in information silos that prevent the formation of a unified digital infrastructure. Efforts such as Mobility Data Specifications are still at early stages and face resistance from legacy systems and institutional inertia [86]. Moving forward, promoting cross-system data standardization and unified interface design will be essential to enhance interoperability.
• Generative AI reliability and sustainability. Despite their powerful generative and causal reasoning capabilities, LLM-based generative agents are prone to illusion risk—wherein models generate plausible but incorrect or misleading outputs—especially under data sparsity or distribution shifts common in urban mobility contexts [87]. This poses important challenges for reliability in safety-critical applications such as emergency traffic management or energy grid control. Moreover, the substantial computational and energy demands of training and deploying large-scale generative models present a sustainability bottleneck, conflicting with the green and low-carbon goals of smart cities [88]. Future efforts should focus on developing energy-efficient architectures, robust uncertainty quantification methods, and hybrid models that integrate symbolic reasoning to mitigate these risks while maintaining performance.

### Societal and ethical dilemmas

As MMIF is increasingly embedded into real-world urban governance systems, it introduces a new layer of societal and ethical risks. These arise primarily from the deep reliance on large-scale, fine-grained data such as travel trajectories, communication logs, and energy usage patterns, which elevates concerns about privacy infringement, data security, and surveillance. Beyond privacy, the growing use of automated, data-driven decision-making mechanisms in areas such as transportation relocation and urban resource allocation exposes systemic vulnerabilities in fairness and transparency. Algorithmic biases embedded in model training or objective functions may result in unequal service distribution, amplifying existing urban inequalities [89]. Moreover, the opaque nature of many AI-driven models limits public understanding and oversight, potentially eroding institutional trust. In the future, introducing ethical evaluation mechanisms and regulatory frameworks will be necessary to ensure the fairness and controllability of model-driven decisions. Beyond privacy and fairness, MMIF must integrate behavioral models [90] (e.g., prospect theory) and participatory governance to address human factors, prevent sociotechnical gaps, and ensure that systems are smart, socially sustainable, and human centric [91].

### Future endeavors

The core objectives of MMIF are to capture complexity, enable anticipation, and support transparent intervention. The further development of MMIF in urban mega-mobility systems will require a reorientation of research priorities toward flexibility, generalizability, and interpretability. This will require rethinking model architectures and research paradigms to accommodate dynamic urban conditions, support autonomous yet explainable control, and enable coordination across interdependent subsystems. The next phase of development will be shaped by these converging priorities: enhancing model adaptability through online learning and transfer mechanisms, leveraging AI for scalable system optimization, advancing theories of explainable emergence, and institutionalizing interdisciplinary collaboration and regulatory co-design to ensure deployment feasibility and societal alignment.

• Enhancing adaptability and generalization capabilities. Future MMIF models will need stronger adaptability and generalization capabilities to cope with the dynamic changes in urban transportation systems. This includes incorporating adaptive modeling, transfer learning, and online optimization mechanisms, enabling models to quickly generalize across different scenarios while reducing reconstruction costs [92]. Additionally, by implementing dynamic feedback mechanisms, models will be able to self-calibrate and optimize in real-time environments [84].
• AI-driven complex system optimization. AI will sustain its critical function in advancing smart urban ecosystems [93]. By leveraging multi-agent distributed reinforcement learning and automated machine learning techniques, MMIF models can achieve autonomous scheduling and resource optimization in large-scale urban environments [94]. Additionally, causal discovery algorithms are becoming increasingly important for identifying hidden mechanisms in urban mega-mobility systems with numerous interacting elements, especially in domains where controlled experiments are impractical. Integrating causal inference with machine learning could enable AI to automatically extract interpretable causal structures from high-dimensional data, offering physically meaningful explanations for otherwise black-box models. This convergence of AI-driven optimization and causal reasoning has the potential to transform our ability to model, predict, and manage complex, interconnected systems in a scientifically rigorous and explainable manner.
• Explainable decision and emergence. In the context of a mega-mobility system with deep coupling of various networks, a fundamental frontier challenge lies in the quantitative characterization of emergent phenomena and causal architectures. Current theoretical explorations include causal emergence criteria based on effective information, partial information decomposition frameworks, and synergetic information clusters in the mega-mobility system [95]. Future breakthroughs may yield universal emergence laws analogous to thermodynamics’ entropy principle, capable of predicting conditions under which autonomous macroscopic regularities emerge. Concurrently, causal discovery algorithms will play a pivotal role in extracting hidden mechanisms from the observational data of urban mobility systems, which involve highly stochastic and socially influenced human behaviors. These systems share key challenges with sociobiological domains, where controlled experimentation is typically infeasible. The synergistic integration of causal inference with machine learning is anticipated to automatically distill interpretable macroscopic causal networks from intricate datasets in the mega-mobility context. This convergence promises to endow black-box models with physics-grounded interpretations, bridging data-driven patterns with first-principles understanding through causal emergence theory [96].
• Interdisciplinary collaboration and policy guidance. Research on urban mobility systems involving multiple networks will increasingly rely on interdisciplinary collaboration and policy guidance. Moving forward, promoting the deep integration of urban science, data science, social science, and policy research will be essential for tackling cross-domain challenges [97]. Additionally, government-led standardization and policy guidance will be necessary to facilitate data sharing, regulate algorithms, and promote technological deployment, thereby enhancing the feasibility and impact of models in real-world management.

## Conclusions

In the era of AI-driven rapid iteration for smart cities, the investigations of urban mega-mobility systems embody a pivotal frontier in 21st-century science. This review article establishes the core paradigm of MMIF, which organically combines the emergent properties at the macrolevel with the behavioral mechanisms of microlevel individuals. By introducing continuous, bidirectional feedback between these scales, MMIF transforms macro- and microperspectives into a self-consistent, co-adaptive modeling cycle, enabling cities to sense, respond, and reorganize dynamically. We hope that the MMIF paradigm will inspire researchers and practitioners to collectively redefine the urban systems: not as a collection of problems to be solved but as a complex living entity to be understood, shaped, and coevolved.
